# Aucubin from Eucommiae Cortex Alleviates Tendinopathy via an Estrogen Receptor β-Mediated Mechanism

**DOI:** 10.3390/ph19020194

**Published:** 2026-01-23

**Authors:** Guorong Zhang, Shuang Wang, Keyi Wu, Meiqi Sun, Qiang Chen, Jialin Wei, Yue Luan, Ye Qiu, Zhidong Qiu

**Affiliations:** 1 School of Basic Medical Sciences, Changchun University of Chinese Medicine, Changchun 130117, China; z_guorong@163.com; 2 School of Pharmacy, Changchun University of Chinese Medicine, Changchun 130117, China; 15804421258@163.com (S.W.); 13154379764@163.com (K.W.); sunmeiqi4222@163.com (M.S.); chenqiang990105@163.com (Q.C.); q435507352@163.com (J.W.); 15843186923@163.com (Y.L.)

**Keywords:** Eucommiae Cortex, aucubin, tendinopathy, estrogen receptor β (ERβ), phytoestrogen, oxidative stress, collagen remodeling

## Abstract

**Background**: Tendinopathy remains a prevalent musculoskeletal disorder with limited disease-modifying pharmacotherapy. This study aimed to identify a reparative agent from the traditional medicinal herb Eucommiae Cortex and elucidate its mechanism of action. **Methods**: A bioactive fraction was first identified through a bioactivity-guided strategy using tenocyte cytoprotection and migration assays, then characterized by UHPLC-HRMS/MS. Its major constituent, aucubin (AU), which mirrors the fraction’s key pharmacological activities, was evaluated both in vitro and in vivo. In H_2_O_2_-injured tenocytes, AU’s effects on viability, apoptosis, oxidative stress (ROS, MDA, SOD) and inflammation (IL-1β, TNF-α) were assessed, with specific focus on estrogen receptor (ER) pathway involvement using pharmacological tools (17β-estradiol and (R, R)-THC). In a collagenase-induced Achilles tendinopathy model using male SD rats, AU’s therapeutic efficacy was evaluated via multimodal assessment: ultrasonography, histopathology (H&E, Masson’s trichrome, Sirius red), TEM, immunohistochemistry, and biochemical analysis of tissue markers. **Results**: AU effectively attenuated H_2_O_2_-induced tenocyte injury by enhancing viability, reducing apoptosis, and mitigating oxidative/inflammatory stress. These effects were mimicked by 17β-estradiol and reversed by the selective ERβ antagonist (R, R)-THC, indicating ERβ dependence. In vivo, AU treatment promoted structural and functional recovery, improved collagen maturity (increased Col I/Col III ratio and fibril diameter), suppressed matrix degradation (MMP-3, MMP-13) and apoptosis, and reduced oxidative stress and inflammation in tendon tissue. **Conclusions**: This study identifies aucubin as a novel phytoestrogenic compound from Eucommiae Cortex that promotes tendon repair through an ERβ-mediated mechanism. These findings position ERβ activation as a promising therapeutic strategy for tendinopathy and highlight AU as a promising lead compound for further development.

## 1. Introduction

Tendinopathy is a prevalent and debilitating musculoskeletal disorder, characterized by activity-related pain, functional impairment, and an increased risk of tendon rupture [1]. Its complex pathogenesis involves a detrimental cascade of inflammation, oxidative stress, dysregulated apoptosis, and disrupted extracellular matrix (ECM) homeostasis, ultimately leading to collagen disorganization and impaired biomechanical function [1,2,3]. Current first-line pharmacotherapies, primarily non-steroidal anti-inflammatory drugs (NSAIDs) and corticosteroid injections, offer only transient symptomatic relief and fail to promote genuine tissue regeneration [4]. Notably, some evidence suggests they may even compromise long-term healing [5]. This highlights a significant unmet clinical need for disease-modifying agents capable of driving high-quality tendon repair.

In the search for novel therapeutic strategies, medicinal plants with a long history of use provide a valuable source of leads. One such example is Eucommiae Cortex, the dried bark of the tree Eucommia ulmoides Oliv., which has been traditionally described as beneficial for both tendons and bones [6]. Modern pharmacology has validated its efficacy against bone diseases such as osteoporosis [7]. Interestingly, this traditional notion finds a compelling parallel in modern biology: tendons and bones share a mesenchymal origin, fibroblast-like cell phenotypes, and a collagen-dominated extracellular matrix. Therefore, we hypothesized that the pharmacological basis for the herb’s established bone-strengthening (anti-osteoporotic) effects might also underpin its potential for tendon repair, and that its constituents may hold promise for the treatment of tendinopathy.

To identify the key constituents responsible for this potential tendon-protective effect, we focused on the major bioactive classes within Eucommiae Cortex. Phytochemical studies highlight lignans and iridoid glycosides as their most characteristic and abundant components, both of which have been implicated in their bone-strengthening effects [7,8]. Iridoid glycosides, in particular, have garnered attention for their broad-spectrum bioactivities relevant to tissue repair, including marked anti-inflammatory effects, promotion of collagen synthesis, and acceleration of wound healing [9,10]. Among these, aucubin (AU), a principal iridoid glycoside, emerges as a particularly promising candidate, warranting a focused investigation into its effects on tendon tissue.

AU’s pharmacological profile is uniquely aligned with the multifaceted pathogenesis of tendinopathy. Extensive research documents its potent anti-inflammatory, antioxidant, and anti-apoptotic properties across various disease models [11,12,13]. Building on the general pro-repair profile of iridoids, AU has been specifically shown to promote collagen synthesis [10]. Most notably, it exhibits phytoestrogenic activity primarily via activation of the estrogen receptor β (ERβ) subtype [14]. This specific ERβ-targeting property is highly relevant to tendon biology. Estrogen signaling plays a crucial role in tendon homeostasis and repair. Clinical and preclinical evidence indicates that estrogen deficiency impedes tendon healing, whereas estrogen supplementation can promote repair processes [15,16,17]. Mechanistically, ERβ has been identified as the predominant estrogen receptor subtype in tendon tissue and is a key positive regulator of tendon repair. Studies using ERβ-knockout models demonstrate that ERβ deficiency leads to impaired early healing (reduced tenocyte proliferation, increased apoptosis, and aberrant tissue composition) and compromised late-stage biomechanical recovery [18,19]. Given that (i) ERβ activation is beneficial for tendon repair, and (ii) AU is reported to act as a phytoestrogen preferentially targeting ERβ, we posited that the ERβ pathway constitutes a plausible and specific mechanistic link through which AU might exert tendon-protective effects. This hypothesis offers a testable molecular pathway to explore the potential cross-talk between bone and tendon therapeutics. Despite these compelling mechanistic links, the therapeutic effects of AU on tendinopathy and its underlying mechanisms remain entirely unexplored.

To address this knowledge gap, we designed a comprehensive study to evaluate the therapeutic potential of AU and its mechanism of action. First, we employed bioactivity-guided fractionation to identify the active fraction of Eucommiae Cortex extract and confirmed AU as a principal cytoprotective component. Second, using an in vitro model of H_2_O_2_-induced oxidative damage in rat primary tenocytes, we systematically investigated AU’s protective effects and its potential dependence on the ERβ pathway, employing the agonist 17β-estradiol (E2) and the selective ERβ antagonist (R, R)-THC [20]. Finally, we evaluated the translational efficacy of AU in a well-established rat Achilles tendinopathy model induced by collagenase I injection [21,22], assessing its impact on histopathological recovery, collagen remodeling, ultrastructural restoration, and the modulation of the local repair microenvironment (e.g., inflammatory factors, oxidative stress, and matrix metabolism). This work aims to identify a novel natural product-derived candidate for tendinopathy treatment and to elucidate its ERβ-mediated mechanism of action.

## 2. Results

### 2.1. The Aqueous Fraction of Eucommiae Cortex Protects Tenocytes from Oxidative Stress and Promotes Migration

From 100 g of Eucommiae Cortex, we prepared a total crude extract. The majority of this extract (about 90%) was further processed through sequential solvent partitioning, yielding four separate fractions based on polarity: petroleum ether (1.09 g), ethyl acetate (0.66 g), n-butanol (2.36 g), and aqueous (6.03 g).

Primary rat tenocytes were used for all assays. Our first step was a cytotoxicity screen to establish safe working concentrations for the four fractions. Screening across a broad range (0.01–2 mg/mL) revealed that the petroleum ether, n-butanol, and aqueous fractions were non-toxic at all tested doses. However, the ethyl acetate fraction proved toxic at the highest concentrations (1–2 mg/mL) (Figure S1). Therefore, in all subsequent functional tests, we chose a uniform safety concentration—less than or equal to 0.5 mg/mL.

**Figure 1:**
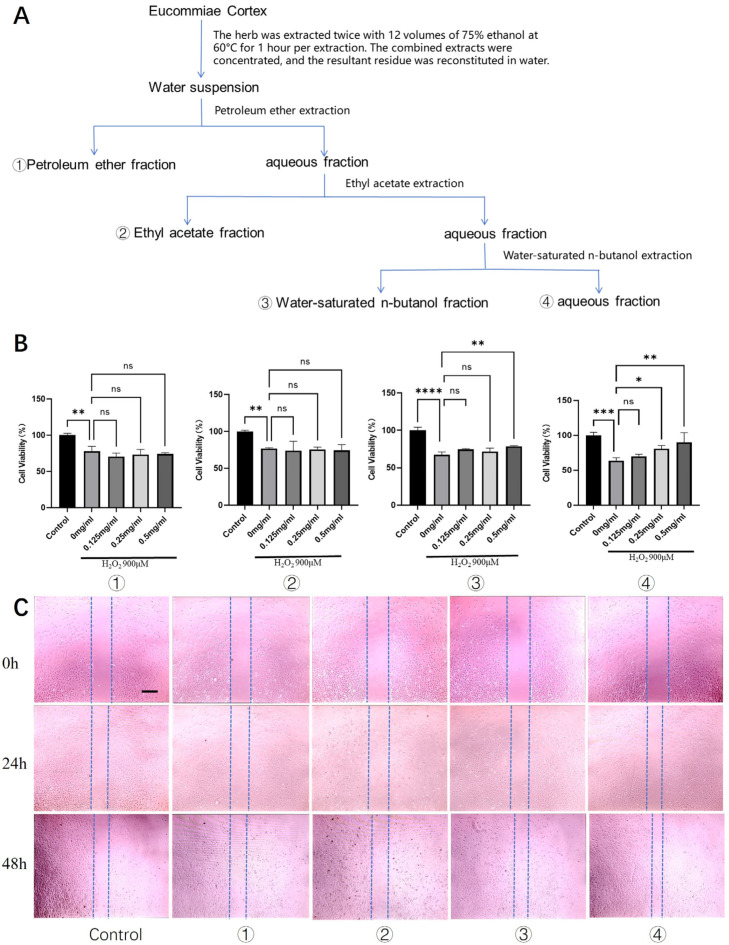
Screening of bioactive fractions from Eucommiae Cortex for cytoprotective and promigratory activities in rat tenocytes. (**A**) Schematic flowchart illustrating the preparation of different Eucommiae Cortex extract fractions. (**B**) Viability of rat primary tenocytes treated with various fractions under H_2_O_2_-induced oxidative stress (*n* = 6). (**C**) Migration capacity of tenocytes following treatment with the different fractions, as assessed by a scratch wound healing assay (*n* = 3). Scale bars: 100 μm. Data are presented as mean ± SD. ns, not significant; * *p* < 0.05, ** *p* < 0.01, *** *p* < 0.001, **** *p* < 0.0001.

To establish an in vitro model of oxidative injury, we optimized the concentration of H_2_O_2_ and determined that 900 μM effectively induced consistent sub-lethal cytotoxicity. We used this model to test the four fractions for cytoprotection. The aqueous fraction stood out, showing the strongest protective effect. It restored tenocyte viability in a dose-dependent manner, with 0.5 mg/mL performing better than 0.25 mg/mL (Figure 1B). The n-butanol fraction at 0.5 mg/mL offered only a mild protective effect.

Furthermore, we assessed the impact on tenocyte migration, a critical process in tendon repair. Scratch wound healing assays revealed that after 24 h, the aqueous fraction began to show a moderate but notable promotion of wound closure compared to the model group. By 48 h, the aqueous fraction had significantly enhanced the healing process, demonstrating the most robust pro-migratory effect among all fractions (Figure 1C). The n-butanol fraction also exhibited a moderate pro-migratory effect at the 48 h time point.

Collectively, these results identified the aqueous fraction of Eucommiae Cortex extract as the most promising active fraction, conferring both protection against oxidative stress and enhancement of migratory capacity in tenocytes.

### 2.2. Identification of Aucubin as a Predominant Constituent and Preliminary Validation of Its Tendon-Protective Effects

**Figure 2:**
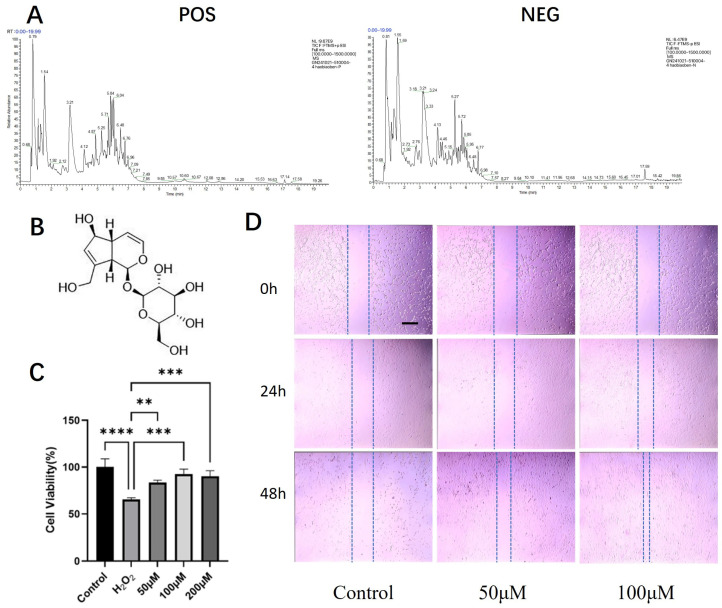
UHPLC-HRMS/MS profiling of the active aqueous fraction and evaluation of aucubin’s effects on tenocytes. (**A**) Total ion chromatogram (TIC) of the aqueous fraction in positive (POS) and negative (NEG) ionization modes. (**B**) Chemical structure of AU. (**C**) Viability of rat primary tenocytes treated with AU under H_2_O_2_-induced oxidative stress (*n* = 6). (**D**) Migration capacity of AU-treated tenocytes, assessed by a scratch wound healing assay (*n* = 3). Scale bars: 100 μm. Data are presented as mean ± SD. ** *p* < 0.01, *** *p* < 0.001, **** *p* < 0.0001.

UHPLC-HRMS/MS analysis of the bioactive aqueous fraction led to the identification of ten major compounds (Table 1, Figure 2A). Aucubin (AU) and geniposidic acid were identified as the two overwhelmingly predominant iridoid glycosides, with peak areas more than ten-fold greater than those of other constituents. AU itself accounted for the most abundant peak.

We prioritized AU for immediate functional validation based on a dual rationale: (1) its status as the most abundant small molecule in the active fraction provided a strong chemical basis; and (2) as reviewed in the Introduction, AU’s reported pharmacological profile—including anti-inflammatory, antioxidant, pro-collagen, and ERβ-modulating activities—directly intersects with the multifaceted pathogenesis of tendinopathy, making it a highly plausible candidate.

The cytotoxicity of AU on rat primary tenocytes was first assessed. The CCK-8 assay confirmed that treatment with AU at concentrations ranging from 6.25 to 400 μM for 24 h did not induce any significant cytotoxicity (Figure S2), defining a safe range for subsequent experiments.

We then evaluated the protective effect of AU against H_2_O_2_-induced oxidative injury. Exposure to 900 μM H_2_O_2_ significantly compromised tenocyte viability. However, co-treatment with AU at 50, 100, and 200 μM conferred substantial protection, each resulting in a statistically significant recovery of cell viability compared to the model group (Figure 2C). Notably, the cytoprotective effects at 100 and 200 μM were significantly stronger than that at 50 μM, while no significant difference was observed between these two higher concentrations, indicating a potential plateau in the efficacy within this dose range.

Furthermore, the potential of AU to enhance tenocyte migration was examined using a scratch wound healing assay. After 24 h, 100 μM AU showed a slight promotive effect on wound closure. This effect became more pronounced at the 48 h time point, where both 50 μM and 100 μM AU significantly accelerated the healing process, with 100 μM AU demonstrating the most robust effect (Figure 2D).

Collectively, the chemical predominance of AU in the active fraction, supported by its mechanistically relevant pharmacological reputation, provided a compelling rationale for its selection. The subsequent confirmation of its non-cytotoxic, antioxidant, and promigratory activities in tenocytes validated this choice and firmly established AU as our lead compound for this study for all subsequent in-depth investigations.

### 2.3. Aucubin Attenuates H_2_O_2_-Induced Oxidative Damage and Inflammation in Rat Tenocytes, Potentially via an Estrogen Receptor Pathway

**Figure 3:**
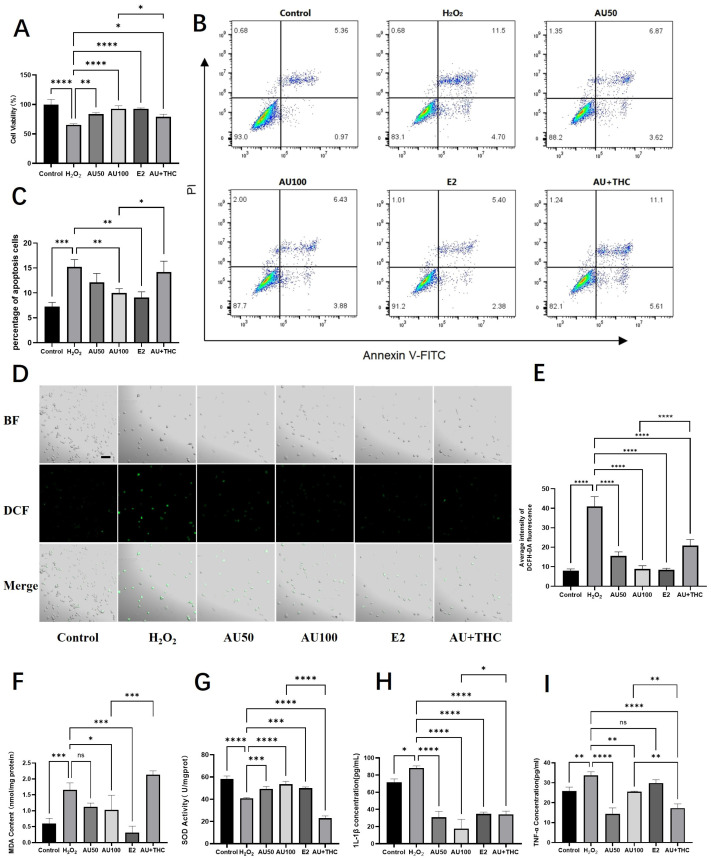
Aucubin attenuates H_2_O_2_-induced oxidative damage and inflammation in rat tenocytes, mechanisms involving the estrogen receptor pathway. (**A**) Viability of tenocytes after treatment (*n* = 6). (**B**) Representative flow cytometry plots of apoptosis. (**C**) Quantitative analysis of apoptotic rates (*n* = 3). (**D**) Representative micrographs of intracellular ROS levels detected by DCFH-DA staining. Scale bars: 100 μm. (**E**) Quantitative analysis of ROS fluorescence intensity (*n* = 3). (**F**–**I**) Effects of AU on (**F**) malondialdehyde (MDA) content, (**G**) superoxide dismutase (SOD) activity, (**H**) IL-1β, and (**I**) TNF-α levels (*n* = 3). Notably, the protective effects of AU on most parameters were mimicked by the estrogen agonist 17β-estradiol (E2) and antagonized by the estrogen receptor antagonist (R, R)-THC. Data are presented as mean ± SD. * *p* < 0.05, ** *p* < 0.01, *** *p* < 0.001, **** *p* < 0.0001.

To further elucidate the cytoprotective mechanism of aucubin (AU), we investigated its effects on oxidative stress parameters and inflammation, with a focus on the potential involvement of the estrogen receptor (ER) pathway. As illustrated in Figure 3A, exposure to 900 μM H_2_O_2_ significantly reduced tenocyte viability to 65.27% compared to the control. Co-treatment with 50 μM and 100 μM AU remarkably restored cell viability to 83.51% and 92.51%, respectively. The protective effect of 100 μM AU was comparable to that of the estrogen agonist 17β-estradiol (E2, 92.65%). Crucially, the addition of the estrogen receptor antagonist (R, R)-THC significantly attenuated the protective effect of 100 μM AU, lowering the viability to 78.90%. This antagonism suggests ER pathway involvement in AU’s action.

We next assessed apoptosis and necrosis using flow cytometry. H_2_O_2_ induction led to a significant increase in apoptotic and necrotic cells (Figure 3B,C). While 50 μM AU showed a decreasing trend, 100 μM AU and E2 significantly suppressed H_2_O_2_-induced cell death. The protective effect of 100 μM AU was again reversed by (R, R)-THC, further supporting the ER-mediated mechanism.

Subsequent analyses on oxidative stress markers provided consistent results. AU (50 and 100 μM) and E2 significantly mitigated the H_2_O_2_-induced intracellular ROS burst (Figure 3D,E). Similarly, AU at 100 μM and E2 significantly reversed the H_2_O_2_-induced elevation in malondialdehyde (MDA), a lipid peroxidation product, while 50 μM AU showed a non-significant trend (Figure 3F). Concurrently, the H_2_O_2_-impaired superoxide dismutase (SOD) activity was significantly restored by 100 μM AU and E2 (Figure 3G). For all these parameters, the beneficial effects of 100 μM AU were effectively counteracted by the ER antagonist.

Finally, we examined the effects on inflammation. H_2_O_2_ stimulation significantly upregulated the pro-inflammatory cytokines IL-1β and TNF-α (Figure 3H,I). Both AU (at 50 and 100 μM) and E2 significantly reduced IL-1β levels. Similarly, both concentrations of AU also significantly lowered TNF-α levels, whereas E2 induced only a non-significant downward trend. The ER antagonist (R, R)-THC notably reversed the suppressive effect of AU on IL-1β. However, the effect on TNF-α regulation appeared more complex, as co-treatment with the antagonist led to a further decrease rather than a reversal.

In summary, AU effectively counteracts H_2_O_2_-induced oxidative damage by reducing ROS, MDA, and cell death, while enhancing antioxidant capacity (SOD activity). Its anti-inflammatory effect is evident in the suppression of IL-1β. Taken together, the observation that AU’s cytoprotective effects are pharmacologically mimicked by E2 and attenuated by the ER antagonist (R, R)-THC supports the hypothesis that the estrogen receptor pathway contributes significantly to AU’s mechanism of action in tenocytes.

### 2.4. Aucubin Promotes Structural Recovery and Improves Collagen Maturity in a Rat Tendinopathy Model

We first established and validated a rat Achilles tendinopathy model via local injections of collagenase I. Successful induction was confirmed by macroscopic observation, which revealed rough tendon surfaces covered with yellowish inflammatory hyperplasia and adhesions to the surrounding tissue (Figure S3B). Histological analysis of H&E-stained sections demonstrated disrupted collagen fiber alignment and a marked infiltration of inflammatory cells in the model group (Figure S3D). The severity of the tendinopathy was further quantified using a histopathological scoring system, which showed a significant elevation in the model group (Figure S3G). Masson’s Trichrome staining revealed loose collagen fibers with a predominant blue hue, indicating poor organization (Figure S3F). Quantitative analysis confirmed a significant increase in the area of blue staining, corresponding to a higher content of immature collagen in the model tendons (Figure S3H). Collectively, these findings confirmed the successful establishment of the tendinopathy model.

**Figure 4:**
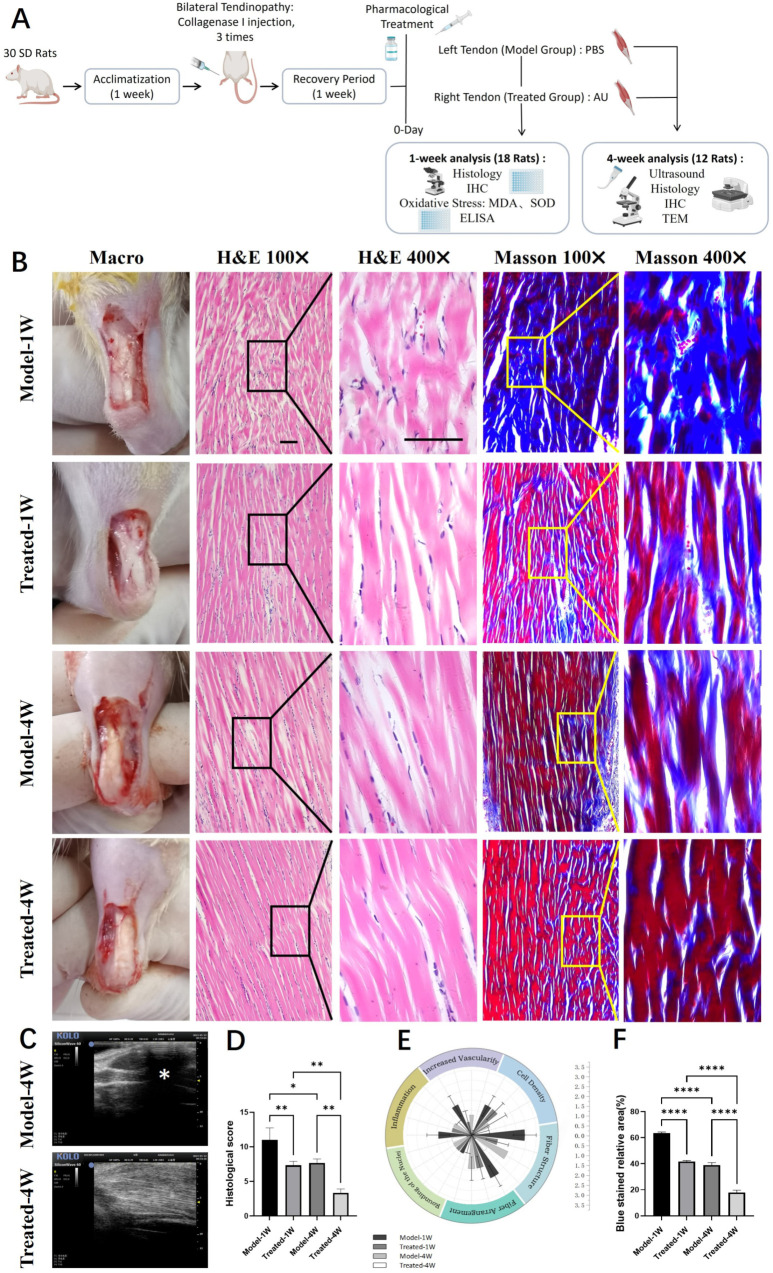
Aucubin ameliorates tendon histopathology and collagen organization in a rat tendinopathy model. (**A**) Schematic diagram of the animal experimental timeline. Schematic diagram was created in BioRender (Zhang, G., 2026; available at https://BioRender.com/klb9yj3). (**B**) Representative macroscopic and histological images of tendon sections stained with H&E and Masson‘s Trichrome. (**C**) Ultrasonographic evaluation of tendon morphology. (**D**) Total histopathological scores based on a modified Movin grading system (H&E-stained sections) (*n* = 6). (**E**) Scores of the six individual parameters from the histopathological evaluation (*n* = 6). (**F**) Quantitative assessment of collagen maturity, shown as the relative area of blue staining (indicative of immature collagen) in Masson’s Trichrome-stained sections (*n* = 6). Scale bar: 100 μm (applies to (**B**)). Data are presented as mean ± SD. * *p* < 0.05, ** *p* < 0.01, **** *p* < 0.0001.

We next evaluated the therapeutic potential of AU over a four-week treatment period. High-frequency small animal ultrasonography was employed to assess tendon morphology in vivo. Tendons in the Model group exhibited focal hypoechoic regions and heterogeneous echotexture (Figure 4C, indicated by *). In contrast, AU-treated tendons displayed more uniform echogenicity and a homogeneous structure, suggesting improved tissue integrity.

Histological examination provided further evidence of AU-mediated repair. Analysis of H&E-stained sections after one and four weeks of treatment showed that AU-treated tendons had better-organized collagen fibers with fewer discontinuities compared to the Model group (Figure 4B). This visual improvement was corroborated by a significant reduction in the total histopathological score, as well as in the scores of all six individual parameters, in the AU-treated group (Figure 4D,E). Masson’s Trichrome staining revealed denser collagen bundles and a noticeable reduction in blue-stained areas in AU-treated tendons, indicating enhanced collagen maturity (Figure 4B). Quantification of the blue area confirmed a significant decrease in the content of immature collagen following AU treatment (Figure 4F).

In summary, AU treatment effectively promoted structural restoration and improved collagen maturity in the rat tendinopathy model, as evidenced by ultrasonography, histopathological scoring, and collagen staining.

### 2.5. Aucubin Improves Collagen Composition and Ultrastructure in a Rat Tendinopathy Model

**Figure 5:**
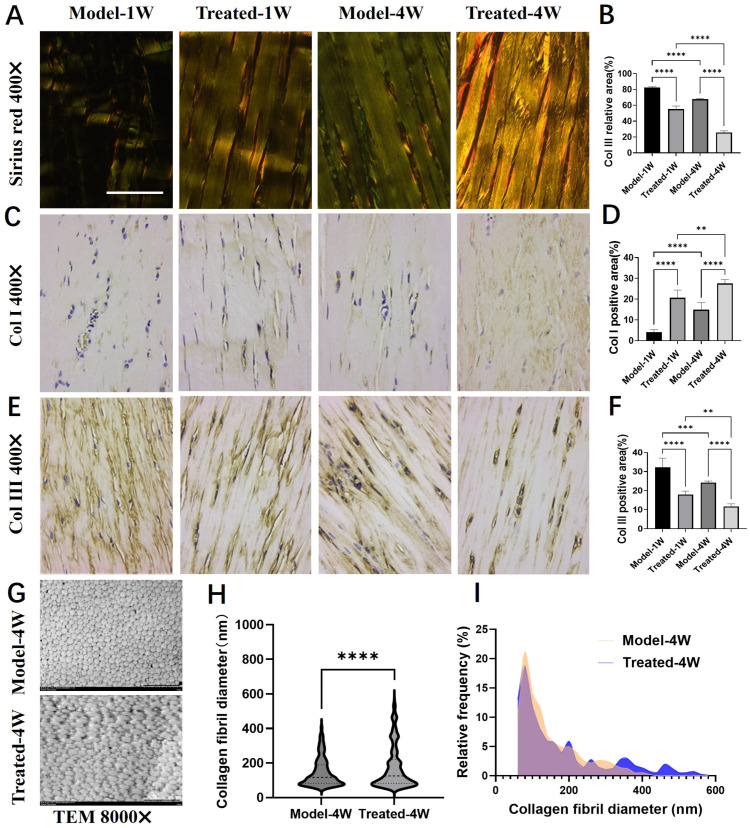
Effects of aucubin on collagen type switching and fibril diameter. (**A**) Representative Sirius Red-stained tendon sections observed under polarized light. (**B**) Quantitative analysis of the relative area of green birefringence, indicative of type III collagen (Col III) (*n* = 6). (**C**,**D**) Immunohistochemical (IHC) analysis of type I collagen (Col I): (**C**) representative images and (**D**) integrated optical density (IOD) quantification (*n* = 6). (**E**,**F**) IHC analysis of Col III: (**E**) representative images and (**F**) IOD quantification (*n* = 6). (**G**) Representative transmission electron microscopy (TEM) images of collagen fibrils. (**H**) Mean collagen fibril diameter and (**I**) fibril diameter distribution across groups (n ≥ 1500). Scale bars: 100 μm (**A**,**C**,**E**); 2 μm (**G**). Data are presented as mean ± SD. ** *p* < 0.01, *** *p* < 0.001, **** *p* < 0.0001.

To assess the impact of AU on collagen remodeling, we first performed Sirius Red staining and examined the sections under polarized light. Collagen type I (Col I) fibers appear orange-red and are thick, whereas collagen type III (Col III) fibers appear green and are thin. At both one and four weeks post-treatment, a more prominent orange-red birefringence was observed in the AU-treated group, whereas the Model group exhibited a predominance of green hue (Figure 5A). Quantitative analysis confirmed a significant reduction in the relative area of green birefringence (indicative of Col III) in the AU-treated group (Figure 5B). Over the course of the experiment, a natural shift towards more mature collagen was observed in both groups, with colors becoming more yellowish at four weeks compared to one week. However, AU treatment significantly accelerated this process.

We next quantified the expression of Col I and Col III via immunohistochemistry (IHC). Consistent with the Sirius Red findings, the natural healing process in the Model group was characterized by a gradual increase in Col I and a decrease in Col III from week 1 to week 4. AU treatment significantly enhanced this shift: the AU-treated group demonstrated a marked upregulation of Col I (Figure 5C,D) and a concurrent downregulation of Col III (Figure 5E,F) at both time points compared to their respective Model groups. These results indicate that AU accelerates tendon repair by promoting the deposition of mature Col I over immature Col III.

Given the critical influence of collagen fibril diameter on the biomechanical properties of tendons [23], we further analyzed the tendon ultrastructure using transmission electron microscopy (TEM). TEM cross-sections revealed the presence of a greater number of large-diameter collagen fibrils in the AU-treated group compared to the Model group (Figure 5G). Quantitative analysis confirmed that AU treatment significantly increased the mean collagen fibril diameter (Figure 5H) and shifted the fibril diameter distribution towards larger sizes (Figure 5I).

In summary, AU treatment favorably modulates the tendon healing process by promoting a mature collagen profile—characterized by a higher Col I/Col III ratio—and facilitating the formation of thicker collagen fibrils, which are essential for restoring tendon strength and function.

### 2.6. Aucubin Modulates Key Pathological Factors and Promotes a Restorative Environment in Tendinopathy Tissue

To further elucidate the mechanism by which AU promotes tendon repair in vivo, we analyzed its effect on key proteases, apoptosis, oxidative stress, and cytokine levels in the tendon tissue.

**Figure 6:**
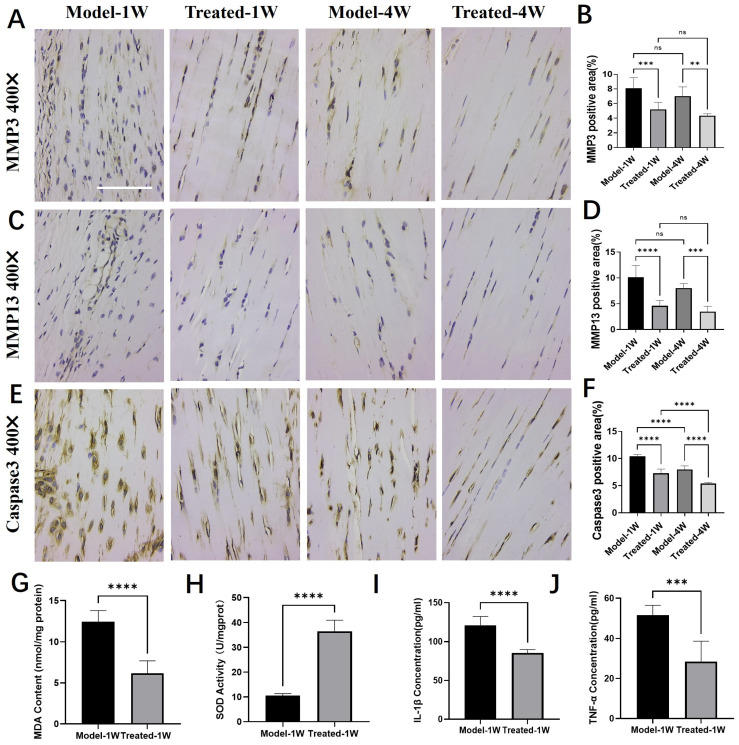
Aucubin regulates the expression of proteases, oxidative stress, and cytokine markers in tendinopathy tissue. (**A**,**B**) Immunohistochemical (IHC) analysis of Matrix Metalloproteinase-3 (MMP-3): (**A**) representative images and (**B**) integrated optical density (IOD) quantification (*n* = 6). (**C**,**D**) IHC analysis of Matrix Metalloproteinase-13 (MMP-13): (**C**) representative images and (**D**) IOD quantification (*n* = 6). (**E**,**F**) IHC analysis of Cleaved Caspase-3: (**E**) representative images and (F) IOD quantification (*n* = 6). (**G**–**J**) Effects of AU on the levels of (**G**) malondialdehyde (MDA), (**H**) superoxide dismutase (SOD) activity, (**I**) IL-1β and (**J**) TNF-α in tendon tissue (*n* = 6). Scale bars: 100 μm (**A**,**C**,**E**). Data are presented as mean ± SD. ns, not significant; ** *p* < 0.01, *** *p* < 0.001, **** *p* < 0.0001.

Immunohistochemical analysis revealed that AU treatment significantly downregulated the expression of key matrix-degrading enzymes and a pro-apoptotic marker. Compared to the Model group, the expression of Matrix Metalloproteinase-3 (MMP-3, Figure 6A,B), Matrix Metalloproteinase-13 (MMP-13, Figure 6C,D), and Cleaved Caspase-3 (Figure 6E,F) was markedly reduced in the AU-treated group at both one and four weeks, indicating a suppression of extracellular matrix degradation and apoptosis.

We next assessed the oxidative stress status in tendon tissue after one week of treatment. AU treatment significantly reduced the level of malondialdehyde (MDA), a lipid peroxidation product (Figure 6G), and concurrently enhanced the superoxide dismutase (SOD) activity, reflecting increased antioxidant capacity (Figure 6H).

Furthermore, AU treatment favorably modulated the cytokine milieu in the injured tendon. ELISA results demonstrated that the levels of the pro-inflammatory cytokines IL-1β (Figure 6I) and TNF-α (Figure 6J) were significantly lower in the AU-treated group than in the Model group.

Collectively, these findings demonstrate that AU orchestrates a multifaceted therapeutic effect in tendinopathy tissue by inhibiting catabolic and pro-apoptotic processes, while alleviating oxidative stress and suppressing inflammation.

## 3. Discussion

Building on the well-documented osteogenic effects reported for extracts of Eucommiae Cortex [6,7] and the shared biological characteristics between bone and tendon tissues, we hypothesized that this medicinal material might also harbor therapeutic potential for tendinopathy. Here, we provide the first comprehensive evidence supporting this hypothesis. We identify aucubin (AU), a predominant iridoid glycoside from Eucommiae Cortex, as an active constituent that confers multi-faceted efficacy against tendinopathy: it protects tenocytes from oxidative stress-induced apoptosis, enhances their migration, promotes structured collagen remodeling in vivo, and orchestrates a restorative tissue microenvironment. Most notably, integrated pharmacological evidence establishes that these therapeutic effects are mediated, at least in part, through an estrogen receptor β (ERβ) mechanism. This finding not only elucidates a key mechanistic pathway but also positions AU as a promising phytoestrogenic candidate for tendon repair.

Our bioactivity-guided fractionation strategy successfully traced the cytoprotective and promigratory activities of the aqueous fraction derived from the ethanolic extract of Eucommiae Cortex to AU, an iridoid constituent whose selection for validation was guided by its chemical predominance and a literature-based prediction of mechanistic relevance to tendinopathy. The protective effects of AU were unequivocal: it significantly restored the viability of tenocytes under oxidative assault (Figure 2C and Figure 3A) and markedly reduced H_2_O_2_-induced apoptosis and necrosis (Figure 3B,C). Mechanistically, this cytoprotection was underpinned by AU’s potent capacity to scavenge ROS, reduce lipid peroxidation (MDA), enhance endogenous antioxidant activity (SOD), and suppress pro-inflammatory cytokines (IL-1β, TNF-α) (Figure 3D–I). These multi-target actions directly counteract the intertwined pathological drivers of tendinopathy—including oxidative stress, apoptosis, and chronic inflammation [1,2,3]. While confirming AU’s broad cytoprotective potential, our work specifically delineates its efficacy and mechanisms within tenocytes, the primary effector cells in tendinopathy. This aligns with and contextually extends AU’s reported protective effects in other cell types [11,12,13].

A pivotal mechanistic insight from this work is the implication of the estrogen receptor β (ERβ) pathway in AU’s action. This conclusion is supported by a consistent pharmacological profile in which AU’s core cytoprotective effects—restoring cell viability (Figure 3A), suppressing apoptosis (Figure 3B,C), mitigating oxidative stress (Figure 3D–G), and reducing IL-1β (Figure 3H)—were mimicked by 17β-estradiol (E2) and, importantly, reversed by the selective ERβ antagonist (R, R)-THC. Although the modulation of TNF-α presented a more complex pattern, the collective data strongly suggest that ERβ signaling plays a major role in the primary mechanism of AU within tenocytes, aligning with its reported phytoestrogenic activity [14].

Importantly, this well-defined cellular mechanism provides a plausible molecular foundation for the multi-faceted therapeutic efficacy of AU, which we subsequently evaluated in vivo using a well-controlled experimental design. First, we exclusively employed male Sprague-Dawley rats to eliminate the confounding effects of the fluctuating endogenous estrogen cycle in females, thereby establishing a stable baseline to clearly attribute any therapeutic effects to AU’s pharmacological action via ERβ. Second, we utilized the rat collagenase-induced Achilles tendinopathy model. While this model simulates an acute injury rather than chronic overuse, it was selected for its high reproducibility and faithful recapitulation of core pathological hallmarks of human tendinopathy, including collagen degradation, MMP overexpression, and inflammatory infiltration [21,24]. Furthermore, our protocol incorporated a 7-day recovery period after the final collagenase injection to allow the resolution of the initial acute inflammatory phase, thereby shifting the therapeutic intervention window to a more persistent, pathology-driven stage. Finally, we adopted a local subcutaneous injection regimen to directly target the injured Achilles tendon, a strategy justified by the limited vascular supply of tendon tissue and employed in other studies of natural product-derived therapeutics [25,26].

Within this optimized model framework, AU treatment effectively translated the cellular benefits into multi-level tissue repair. At the gross and imaging level, AU-treated tendons exhibited improved structural integrity, as evidenced by more uniform echogenicity on ultrasound compared to the heterogeneous, hypoechoic areas in the Model group (Figure 4C). Histologically, AU treatment led to a significant reduction in total histopathological scores (Figure 4D,E) and promoted better-organized collagen fiber architecture in both H&E and Masson’s Trichrome stains (Figure 4B) [27]. Quantification of Masson’s staining confirmed a significant decrease in the area of immature collagen (blue staining) following AU treatment (Figure 4F). This local AU administration effectively promoted substantial morphological and organizational recovery, offering a potential therapeutic strategy distinct from current palliative options like NSAIDs or corticosteroid injections, which fail to promote regeneration and may carry risks [5].

Beyond general tissue repair, AU promoted a qualitatively superior form of healing characterized by enhanced collagen maturity, which is central to functional recovery. The therapeutic correction of an aberrant collagen composition is a key goal in tendinopathy, given that human tendinopathic tissue is characterized by an excessive production of immature type III collagen over mature type I collagen [28]. Our data demonstrate that AU effectively addresses this pathological hallmark. Sirius Red staining under polarized light revealed that AU treatment significantly increased the proportion of mature, thick collagen type I (orange-red birefringence) relative to immature collagen type III (green birefringence) (Figure 5A,B). This shift towards a mature collagen profile was corroborated by immunohistochemistry, showing upregulated Col I and downregulated Col III expression (Figure 5C–F). As the primary structural component of tendon (60–85% dry weight), the restoration of a high Col I/Col III ratio is critical, as type III collagen deposited during early healing forms a disorganized, biomechanically inferior “patch” that persists in tendinopathy [27]. Most importantly, at the ultrastructural level, transmission electron microscopy demonstrated that AU treatment significantly increased the mean diameter of collagen fibrils and shifted the fibril diameter distribution towards larger sizes (Figure 5G–I), which is directly associated with superior biomechanical strength [29]. These findings collectively indicate that AU not only restores collagen content but critically reverses a key pathological feature. By promoting a mature collagen composition (elevated Col I/Col III ratio), AU facilitates the assembly of a higher-quality extracellular matrix, as evidenced by the significant increase in collagen fibril diameter—an ultrastructural hallmark of superior biomechanical strength. This coordinated improvement steers the healing process away from scar formation towards a more regenerative outcome.

To elucidate the molecular underpinnings of this improved repair, we analyzed key regulators of the tendon microenvironment. AU treatment created a more anabolic and less catabolic milieu. It significantly suppressed the expression of matrix-degrading enzymes (MMP-3 and MMP-13) and the pro-apoptotic marker Cleaved Caspase-3 (Figure 6A–F). Elevated caspase-3 is a feature of degenerative human and overload-induced rat tendinopathy [30], and its reduction by AU underscores an anti-apoptotic effect. Given that MMP-3 and MMP-13 can degrade multiple extracellular matrix components, including collagen, and mediate the development of painful tendinopathy [31], their downregulation likely contributes directly to the observed increase in collagen fibril diameter and the shift towards a mature Col I/Col III ratio. This effect may be both a direct consequence of AU’s action and indirectly mediated through its anti-inflammatory and antioxidant properties. Concurrently, the mitigation of oxidative stress (reduced MDA, enhanced SOD activity) and inflammation (lowered IL-1β and TNF-α) within the tendon tissue (Figure 6G–J) supports a pro-regenerative niche by removing key pathological drivers known to exacerbate tissue damage and impair healing. Therefore, AU orchestrates a multifaceted restorative environment by coordinately inhibiting matrix degradation, suppressing apoptosis, and mitigating oxidative stress and inflammation.

While this study provides a multi-faceted characterization of AU’s tendon-reparative effects, several limitations should be acknowledged to guide future research. First, regarding the animal model, the collagenase-induced tendinopathy model, despite our inclusion of a recovery period, simulates an acute injury rather than the chronic mechanical overload typical of human disease. Future studies in validated chronic overuse models are essential to confirm AU’s efficacy under more etiologically relevant conditions. Second, while we documented robust improvements at histological, biochemical, and ultrastructural levels, our study lacks direct biomechanical or functional assessments. Conclusive evidence of true functional recovery requires complementary evaluations, such as measuring the ultimate tensile strength of healed tendons or performing functional gait analysis. Third, concerning mechanistic specificity, our data robustly implicate the ERβ pathway as a central, though not necessarily exclusive, mediator of AU’s effects. The consistent reversal by the selective antagonist (R, R)-THC establishes a strong pharmacological dependency. However, tendon repair is a complex process likely involving crosstalk between multiple signaling pathways. Future studies employing genetic approaches (e.g., tenocyte-specific ERβ knockdown) are warranted not only to confirm its non-redundant role but also to map its interaction with other repair-relevant pathways (e.g., TGF-β, IGF-1), thereby delineating the broader mechanistic network through which AU operates. Looking toward therapeutic translation, future studies should define the local pharmacokinetics of AU within tendon tissue to optimize dosing regimens for topical or injectable delivery. Furthermore, the potential synergistic effects of AU with other constituents of Eucommiae Cortex, such as geniposidic acid [32,33,34,35], warrant exploration to fully appreciate the holistic efficacy of the crude extract.

## 4. Materials and Methods

### 4.1. Preparation of Eucommiae Cortex Extract

Eucommiae Cortex (harvested in May of the current year in Sichuan Province, Mianyang, China) was used as the starting material. The cork layer was removed, and the bark was ground into a coarse powder. A 100 g aliquot of this powder was subjected to extraction. The coarse powder was first soaked in 75% aqueous ethanol at a solvent-to-material ratio of 12:1 (*v*/*w*) for 2 h. To minimize thermal degradation of labile constituents, the extraction was then carried out under gentle reflux, with the temperature controlled at 60 °C using a water bath. We repeated this extraction step twice, each time for 1 h. After combining all the extracts, the mixture was filtered. The filtrate was concentrated by rotary evaporation under reduced pressure at 60 °C until the distinct smell of ethanol was no longer perceptible. Finally, the solution was concentrated to a volume of roughly 80–90 mL.

The concentrate was then diluted with distilled water to a total volume of 100 mL. A 10 mL aliquot of this solution was reserved as the total crude extract. The remaining 90 mL was sequentially partitioned with three solvents: petroleum ether, ethyl acetate, and water-saturated n-butanol. This process yielded four distinct fractions: ① the petroleum ether-soluble fraction, ② the ethyl acetate-soluble fraction, ③ the water-saturated n-butanol fraction, and ④ the aqueous fraction. The complete workflow is outlined in Figure 1A. Each of these four fractions was then concentrated by rotary evaporation at 60 °C under reduced pressure. The concentrates were transferred to evaporating dishes and left to dry completely in a fume hood.

### 4.2. Cell Culture

Primary tenocytes were isolated from the Achilles tendons of Sprague-Dawley rats. The tendons were aseptically dissected, and the surrounding paratenon tissue was meticulously removed. The clean tendon tissues were then minced and digested with type I collagenase (2 mg/mL) for 4 h at 37 °C to release the cells. Following digestion, the isolated tenocytes were cultured in Dulbecco’s Modified Eagle Medium (DMEM; Gibco, Grand Island, NY, USA) supplemented with 10% fetal bovine serum (FBS; ClarkBio, Shanghai, China) and 1% penicillin-streptomycin (HyClone, Logan, UT, USA). The cells were maintained at 37 °C in a humidified incubator with 5% CO_2_. All experiments were conducted using cells from P2 to P4.

### 4.3. Cell Viability Assay

#### 4.3.1. General Procedure

Cell viability was assessed using the Cell Counting Kit-8 (CCK-8; Invigentech, Irvine, CA, USA). Rat primary tenocytes were seeded in 96-well plates at a density of 5 × 10^3^ cells per well and allowed to adhere for 24 h. The cells were then subjected to various treatment conditions as detailed in the subsequent sections. Following the treatment period, the culture medium was replaced with fresh medium containing 10% (*v*/*v*) CCK-8 reagent. The plates were incubated at 37 °C for 1 h, and the absorbance of each well was measured at 450 nm using a microplate reader (Molecular Devices, San Jose, CA, USA).

#### 4.3.2. Screening of Eucommiae Cortex Extract Fractions

To screen the protective effects of different extract fractions, the cells were divided into the following groups:

- Control group: Cultured in H-DMEM complete medium only.
- Model group: Treated with 900 μM H_2_O_2_ (Sigma-Aldrich, St. Louis, MO, USA; Catalog: 88597-100ML-F) to induce oxidative stress injury.
- Experimental groups: Co-treated with 900 μM H_2_O_2_ and one of the Eucommiae Cortex extract fractions (①, ②, ③, or ④) at concentrations of 0.125, 0.25, or 0.5 mg/mL.

#### 4.3.3. Dose-Dependent Effect of Aucubin

To evaluate the dose-dependent effect of aucubin (Yuanye Bio-Technology Co., Ltd., Shanghai, China; Catalog: B21238), the cells were assigned to the following groups:

- Control group: H-DMEM complete medium.
- Model group: 900 μM H_2_O_2_.
- Experimental groups: Co-treated with 900 μM H_2_O_2_ and aucubin at concentrations of 50, 100, or 200 μM.

#### 4.3.4. Mechanistic Investigation with Aucubin and Estradiol

To further investigate the protective mechanism, an experiment involving a positive control and an antagonist was conducted with the following groups:

- Control group: Culture medium without H_2_O_2_.
- Model group (H_2_O_2_): 900 μM H_2_O_2_.
- Experimental group 1 (AU50): 900 μM H_2_O_2_ + 50 μM aucubin.
- Experimental group 2 (AU100): 900 μM H_2_O_2_ + 100 μM aucubin.
- Positive control group (E2): 900 μM H_2_O_2_ + 10 nM 17β-estradiol (E2; Yuanye Bio-Technology Co., Ltd., Shanghai, China; Catalog: B24215).
- Antagonist group (AU+THC): 900 μM H_2_O_2_ + 100 μM aucubin + 200 nM (R, R)-Tetrahydrocannabinol ((R, R)-THC; Tocris Bioscience, Abingdon, Oxfordshire, UK; Catalog:1990/10).

### 4.4. Scratch Wound Healing Assay

#### 4.4.1. General Procedure

The migratory capacity of rat primary tenocytes was evaluated using a scratch wound healing assay. Cells were seeded in 12-well plates at a density of 1 × 10^5^ cells per well and allowed to form a confluent monolayer over 24 h. A standardized wound was then created in each well by scraping the monolayer with a sterile 200 μL pipette tip. After scratching, the cells were gently washed to remove debris and incubated with the respective treatment media, as specified in the sections below. To minimize the influence of cell proliferation, all treatment media were prepared with complete medium supplemented with 0.4% FBS. The wound areas were photographed under an inverted microscope at 0, 24, and 48 h post-scratching.

#### 4.4.2. Screening of Eucommiae Cortex Extract Fractions

To screen the effects of different extract fractions on cell migration, the cells were divided into the following groups after the creation of the scratch:

- Control group: Medium containing 0.4% FBS without any drug.
- Experimental groups: Medium containing 0.4% FBS and one of the Eucommiae Cortex extract fractions (①, ②, ③, or ④) at a concentration of 0.5 mg/mL.

#### 4.4.3. Effect of Aucubin on Cell Migration

To investigate the effect of the key compound aucubin on cell migration, a separate experiment was conducted with the following groups:

- Control group: Medium containing 0.4% FBS without any drug.
- Experimental groups: Medium containing 0.4% FBS and aucubin at concentrations of 50 μM or 100 μM.

### 4.5. Chemical Profiling by Ultra-High-Performance Liquid Chromatography-High Resolution Tandem Mass Spectrometry (UHPLC-HRMS/MS)

The chemical composition of the aqueous fraction of Eucommiae Cortex extract was characterized using ultra-high-performance liquid chromatography coupled with high-resolution tandem mass spectrometry (UHPLC-HRMS/MS; Thermo Fisher Scientific, Waltham, MA, USA). Briefly, 88.4 mg of the sample was homogenized with 1 mL of 70% methanol using an automated grinding system. After vortexing and centrifugation at 12,000× *g* rpm for 10 min at 4 °C, the supernatant was filtered through a 0.22-μm membrane. An internal standard (2-chlorophenylalanine, final concentration 1 mg/L) was added prior to UHPLC-HRMS/MS analysis.

The analysis was performed on a Thermo Vanquish UHPLC system coupled to a Q Exactive HF hybrid quadrupole-Orbitrap mass spectrometer (Thermo Fisher Scientific, Waltham, MA, USA). Chromatographic separation was achieved on a Zorbax Eclipse C18 column (100 mm × 2.1 mm, 1.8 μm) maintained at 30 °C. The mobile phase consisted of (A) 0.1% formic acid in water and (B) acetonitrile, with a flow rate of 0.3 mL/min. A gradient elution program was applied as detailed in Supplementary Table S1. The injection volume was 2 μL.

Mass spectrometry detection was conducted in both positive and negative ionization modes. Key parameters were heater temperature, 325 °C; sheath gas flow rate, 45 arb; auxiliary gas flow rate, 15 arb; sweep gas flow rate, 1 arb; spray voltage, 3.5 kV; capillary temperature, 330 °C; and S-Lens RF level, 55%. Full MS scans (*m*/*z* range 100–1500) were acquired at a high resolution of 120,000 (at *m*/*z* 200), followed by data-dependent MS^2^ scans (dd-MS2, Top 5) at a resolution of 60,000 using higher-energy collisional dissociation (HCD).

Raw data were processed using Compound Discoverer 3.3 software for peak alignment, peak picking, and feature extraction. Compound identification was performed by matching the acquired MS^2^ spectra against the Thermo mzCloud online database and a custom local database (Thermo mzValut).

### 4.6. Analysis of Apoptosis and Necrosis by Flow Cytometry

The rates of apoptosis and necrosis in rat primary tenocytes were determined using an Annexin V-FITC/Propidium Iodide (PI) Apoptosis Detection Kit (Simu Bio-Technology Co., Ltd., Tianjin, China; Catalog: A5001-02P-L). Cells in the logarithmic growth phase were seeded in 6 cm culture dishes at a density of 5 × 10^5^ cells/dish and allowed to adhere for 24 h.

The cells were then divided into the same treatment groups as described in Section 4.3.4. Following 24 h of treatment, the cells were harvested. Briefly, the cells were trypsinized using trypsin solution without EDTA (Solarbio, Beijing, China; Catalog: T1350), washed twice with ice-cold PBS, and resuspended in 1X Binding Buffer (from the kit) at a density of 1 × 10^6^ cells/mL. A 100 μL aliquot of the cell suspension was transferred to a flow cytometry tube and stained with 5 μL of Annexin V-FITC and 5 μL of PI solution. The tubes were gently vortexed and incubated for 15 min at room temperature in the dark. Subsequently, 400 μL of 1X Binding Buffer was added to each tube prior to analysis. All samples were analyzed using a flow cytometer (BECKMAN COULTER, Brea, CA, USA; Serial: A00-1-1102) within 1 h of staining.

### 4.7. Measurement of Intracellular Reactive Oxygen Species (ROS)

Intracellular ROS levels in rat primary tenocytes were measured using the fluorescent probe 2′,7′-dichlorodihydrofluorescein diacetate (DCFH-DA) with a commercial ROS Assay Kit (Beyotime Biotechnology, Shanghai, China; Catalog: S0033S). Cells in the logarithmic growth phase were seeded at a density of 5 × 10^4^ cells per dish into the center of confocal laser dishes and allowed to adhere for 24 h.

The cells were then assigned to the same treatment groups as detailed in Section 4.3.4. After 24 h of treatment, the culture medium was removed. The cells were washed and subsequently incubated with 1 mL of serum-free medium containing 10 μM DCFH-DA at 37 °C for 20 min in the dark. Following incubation, the cells were gently washed three times with serum-free medium to remove any residual probe.

The intracellular fluorescence intensity was immediately observed and captured using a confocal laser scanning microscope (Zeiss LSM 980, Carl Zeiss Microscopy GmbH, Jena, Germany; excitation wavelength: 488 nm, emission wavelength: 525 nm). The captured images were analyzed using ImageJ 1.54f software to quantify the relative ROS levels based on the mean fluorescence intensity.

### 4.8. Measurement of Malondialdehyde (MDA) Content

#### 4.8.1. In Cell Culture

The lipid peroxidation level in rat primary tenocytes was evaluated by measuring the content of malondialdehyde (MDA) using a commercial Micro MDA Assay Kit (Nanjing Jiancheng Bioengineering Institute, Nanjing, China; Catalog: A003-2). Tenocytes were seeded in 6 cm culture dishes at a density of 5 × 10^5^ cells/dish and allowed to adhere for 24 h. The cells were then assigned to the same treatment groups as detailed in Section 4.3.4 and treated for 24 h. After treatment, the cells were trypsinized, collected by centrifugation at 1000× *g* rpm for 10 min, and washed with PBS. The cell pellet was resuspended in 200 μL of PBS and lysed by ultrasonication on ice (300 W output, 5 cycles of 3–5 s sonication with 30 s intervals). The lysate was then centrifuged, and the resulting supernatant was collected for immediate assay. The protein concentration of the supernatant was determined using a BCA Protein Assay Kit (Beyotime Biotechnology, Shanghai, China; Catalog: P0010S) to normalize the MDA values. The MDA content was measured according to the manufacturer’s instructions, and the concentration was expressed as nmol per mg of protein.

#### 4.8.2. In Tendon Tissue Homogenate

The MDA content in Achilles tendon tissue was assessed to evaluate in vivo lipid peroxidation, using the same Micro MDA Assay Kit. Tendon samples were weighed and homogenized in PBS at a ratio of 10 μL per mg of tissue using an electric homogenizer. The homogenate was then centrifuged at 2500× *g* rpm for 20 min, and the resulting supernatant was collected for analysis. The protein concentration was determined using the same BCA Protein Assay Kit for normalization. The MDA content in the tissue supernatant was measured identically to the cellular assay.

### 4.9. Measurement of Superoxide Dismutase (SOD) Activity

#### 4.9.1. In Cell Culture

The antioxidant capacity of rat primary tenocytes was evaluated by measuring the superoxide dismutase (SOD) activity using a WST-1-based commercial SOD Assay Kit (Nanjing Jiancheng Bioengineering Institute, Nanjing, China; Catalog: A001-3). The cell processing method is the same as that in Section 4.8.1. The protein concentration was determined using the same BCA Protein Assay Kit for normalization. The SOD activity in the supernatant was determined strictly according to the manufacturer’s instructions, and the activity was expressed as U per mg of protein.

#### 4.9.2. In Tendon Tissue Homogenate

The SOD activity in Achilles tendon tissue was assessed to evaluate the in vivo antioxidant status, using the same commercial SOD Assay Kit as for the cell culture experiments. The sample processing method is the same as that in Section 4.8.2. The SOD activity was measured according to the manufacturer’s instructions.

### 4.10. Measurement of Cytokine and Growth Factor Levels by ELISA

#### 4.10.1. In Cell Culture

The intracellular levels of inflammatory cytokines in rat primary tenocytes were quantified using commercial enzyme-linked immunosorbent assay (ELISA) kits. Cells in the logarithmic growth phase were seeded in 6-well plates at a density of 2 × 10^5^ cells per well and allowed to adhere for 24 h. The cells were then assigned to the same treatment groups as detailed in Section 4.3.4 and treated for 24 h. After treatment, the cells were trypsinized, collected by centrifugation, washed with PBS, and the cell pellet was resuspended in 200 μL of PBS and lysed by ultrasonication on ice. The lysate was centrifuged, and the supernatant was collected for immediate analysis.

The concentrations of interleukin-1β (IL-1β) and tumor necrosis factor-α (TNF-α) in the supernatant were determined using specific Rat IL-1β (proteintech, Wuhan, China; Catalog: KE20005) and Rat TNF-α (proteintech, Wuhan, China; Catalog: KE20001) ELISA kits, respectively, strictly in accordance with the manufacturers’ instructions. The absorbance was measured using a microplate reader, and the cytokine concentrations were calculated based on the standard curves and normalized to the total protein concentration.

#### 4.10.2. In Tendon Tissue Homogenate

The levels of inflammatory cytokines in Achilles tendon tissue were quantified using the same ELISA methodology. Tendon samples were weighed and homogenized in PBS at a ratio of 20 μL per mg of tissue. The homogenate was centrifuged at 2500× *g* rpm for 20 min, and the resulting supernatant was collected.

The concentrations of IL-1β and TNF-α were determined using the same aforementioned kits. All assays were performed following the manufacturers’ protocols, consistent with the cellular analysis. The final concentrations for all analytes were expressed as pg per mL.

### 4.11. Animal Modeling and Drug Administration

A total of thirty specific pathogen-free (SPF), eight-week-old male Sprague-Dawley (SD) rats were used in a self-controlled study design [25]. Only male rats were used to control for the potential confounding effects of fluctuating endogenous estrogen levels. No genetic modifications or prior procedures were involved. This design yielded a final sample size of *n* = 6 tendons per group (Model vs. Treated) for each specific assay at each time point. All animals were purchased from Liaoning Changsheng Biotechnology Co., Ltd. (Benxi, China; License No.: SCXK (Liao) 2020-0001). The experimental protocol was approved by the Animal Ethics Committee of Changchun University of Chinese Medicine (Approval No.: 2023143). The overall experimental timeline is schematically illustrated in Figure 4A.

After one week of acclimatization, a bilateral tendinopathy model was established. Under brief anesthesia, 50 μL of collagenase I solution (2 mg/mL) was injected into the midpoint of both Achilles tendons per rat. This injection was repeated every other day for a total of three injections, followed by a 7-day recovery period to allow for the resolution of the acute inflammatory phase.

Post-recovery, daily subcutaneous pharmacological treatment was initiated to maximize local drug delivery to the poorly vascularized tendon tissue. For each animal, the left tendon served as the Model group and received 75 μL of PBS. The right tendon served as the Treated group and received 75 μL of PBS containing 200 μM aucubin (AU). The dosage of the drug was obtained through preliminary experiments.

#### 4.11.1. Sample Collection and Analysis at One Week

After one week of treatment, 18 rats were euthanized by an overdose of sodium pentobarbital. Bilateral Achilles tendons were harvested (yielding *n* = 18 Model and *n* = 18 Treated samples) for the following analyses: Histological examination with Hematoxylin and Eosin (H&E), Masson’s Trichrome, and Sirius Red staining. Immunohistochemical (IHC) analysis of target proteins. Oxidative stress assessment by measuring MDA content and SOD activity. Cytokine factor measurement using ELISA.

#### 4.11.2. Sample Collection and Analysis at Four Weeks

The remaining 12 rats received four weeks of continuous treatment. Prior to euthanasia, tendon repair was assessed using a high-frequency small animal ultrasound imaging system. Subsequently, bilateral tendons were harvested (yielding *n* = 12 Model and *n* = 12 Treated samples) for: Histological examination (H&E, Masson’s Trichrome, Sirius Red). Immunohistochemical (IHC) analysis. Ultrastructural analysis of collagen fibrils using transmission electron microscopy (TEM).

### 4.12. Assessment of Tendon Repair by High-Frequency Ultrasound Imaging

The in vivo evaluation of Achilles tendon repair was conducted using a high-frequency small animal ultrasound imaging system (Kolo Medical Technology Co., Ltd., Suzhou, China; Model: Silicon Wave 60). The operating center frequency of this system is 30 MHz, which enables the provision of high-resolution images and facilitates detailed morphological assessment.

Before imaging, the rats were first anesthetized and the hair around the Achilles tendon area was removed using depilatory cream to ensure the best acoustic coupling effect. Then the rats were placed in a prone position with their hind limbs extended. A suitable amount of ultrasound coupling agent was applied to the exposed skin. The ultrasound probe was placed along the longitudinal axis of the Achilles tendon and scanned from the proximal end of the tendon-tendon membrane junction to the distal end of the calcaneal insertion point. Ultrasound images were taken to evaluate the structural integrity and repair status of the Achilles tendon.

### 4.13. Morphological and Histological Analysis

After performing euthanasia, the Achilles tendons were exposed and photographed to document their macroscopic morphological features. Six tendon samples were selected from each group for histological processing: fixation, paraffin embedding, and cutting into 4–5 μm thick sections.

Consecutive sections were stained for specific assessments. H&E staining was used to evaluate the overall tissue structure and cell morphology. Histopathological scoring was performed in a blinded manner by two experienced observers using a modified Movin grading system (Table S2) [36]. This system quantified six different parameters ranging from 0 (normal) to 3 (the most severe abnormality), and the average score of the two observers was used for comparison between groups.

Masson’s Trichrome staining was used for the quantitative assessment of collagen maturity; the area fraction of the blue-stained, indicative of immature collagen, was measured as a percentage of the total tissue area using ImageJ software. Sirius Red staining was used to further characterize collagen organization and maturity. Under polarized light, thicker and more mature type I collagen fibers exhibited intense orange-red birefringence, while thinner and less mature type III collagen fibers appeared green. To quantify collagen maturity, the area fraction of green birefringence (representing type III collagen) was measured as a percentage of the total birefringent area using ImageJ software.

### 4.14. IHC Analysis

The paraffin-embedded tendon sections were subjected to immunohistochemical staining to observe the expression and localization of key proteins related to tendon lesions. The staining was performed using an IHC kit (Servicebio, Wuhan, China; Catalog: G1212) and specific primary antibodies from Servicebio Co., Ltd., Wuhan, China against the following targets: Collagen Type I (Col I; Catalog: GB11022-3), Collagen Type III (Col III; Catalog: GB111629), Matrix Metalloproteinase-3 (MMP-3; Catalog: GB11131), Matrix Metalloproteinase-13 (MMP-13; Catalog: GB11247), and Cleaved Caspase-3 (Catalog: GB11532).

After standard deparaffinization, rehydration, and antigen retrieval steps, tissue sections were incubated with specific primary antibodies at 4 °C overnight. Following PBS washes, sections were treated with an HRP-conjugated Goat Anti-Rabbit IgG secondary antibody (Servicebio; Cat# GB23303) for 50 min at room temperature. Antigen signal was developed using DAB chromogen, and nuclei were counterstained with hematoxylin. Stained sections were imaged under a light microscope. Protein expression levels were quantified by analyzing the intensity and area of the DAB brown signal.

### 4.15. Analysis of Ultrastructure by TEM

Tendon ultrastructure was examined by transmission electron microscopy (TEM; Hitachi High-Tech Science Corporation, Tokyo, Japan). Aiming to minimize mechanical artifacts, tendon samples were processed for fixation within 1–3 min after dissection. The tissue was immediately placed in primary fixative and trimmed into small cubes of approximately 1 mm^3^. This was followed by standard processing steps: post-fixation, dehydration, and embedding. Subsequently, ultrathin Sections (60–80 nm in thickness) were cut, stained, and imaged. For quantitative analysis, we measured the cross-sectional diameters of at least 1500 collagen fibrils per group from comparable regions using ImageJ software. Here, the diameter of a single fibril was taken as the maximum measurable distance across its profile.

### 4.16. Statistical Analysis

Data are presented as mean ± standard deviation (SD). The sample size (n) for each experiment, detailed in figure legends, was based on common practice in comparable tendinopathy studies. Statistical analyses were performed using GraphPad Prism (v9.0). All datasets were assessed for normality and homogeneity of variances. Comparisons between two groups used an unpaired, two-tailed Student’s *t*-test (or Mann–Whitney U test for non-parametric data). Multiple group comparisons used one-way ANOVA followed by Tukey’s test (or Kruskal–Wallis with Dunn’s test for non-parametric data). A *p*-value < 0.05 was considered significant.

## 5. Conclusions

In conclusion, this study systematically identifies aucubin (AU), a major iridoid glycoside from Eucommiae Cortex, as a promising therapeutic candidate for tendinopathy. Through a sequential strategy of bioactivity-guided fractionation, chemical characterization, and multi-level biological evaluation, we demonstrate that AU confers comprehensive efficacy: it protects tenocytes by mitigating oxidative stress, inflammation, and apoptosis primarily through an estrogen receptor β (ERβ)-mediated mechanism, and translates these cellular benefits into the promotion of high-quality, mature collagen remodeling and restoration of tissue homeostasis in vivo. The demonstrated involvement of the ERβ pathway in AU’s effects highlights it as a key mechanistic contributor and positions AU as a promising phytoestrogenic lead compound. These findings provide a solid pharmacological foundation for the further development of AU and highlight the potential of modulating ERβ signaling as a rational strategy for innovative tendon repair therapies.

## Figures and Tables

**Table 1:** Chemical constituents identified in the aqueous fraction of Eucommiae Cortex extract by UHPLC-HRMS/MS.

No.	Name	Formula	RT (min)	Area	Class	POS/NEG
1	Aucubin	C_15_ H_22_ O_9_	1.678	41,964,455,181.2018	Iridoid Glycoside	NEG
2	Geniposidic acid	C_16_ H_22_ O_10_	3.398	20,541,473,178.9968	Iridoid Glycoside	NEG
3	Pinoresinol diglucoside	C_32_ H_42_ O_16_	5.979	2,506,560,341.6169	Lignan Glycoside	NEG
4	Vanillic Acid	C_8_ H_8_ O_4_	3.09	1,279,262,095.7745	Phenolic Acid	POS
5	(+)-Pinoresinol	C_20_ H_22_ O_6_	5.723	313,375,959.2661	Lignan	POS
6	Geniposide	C_17_ H_24_ O_10_	6.075	148,298,126.9537	Iridoid Glycoside	NEG
7	Caffeic acid	C_9_ H_8_ O_4_	5.799	127,184,420.6808	Phenylpropanoid	POS
8	Chlorogenic acid	C_16_ H_18_ O_9_	5.383	90,675,512.1617	Phenylpropanoid	NEG
9	Ferulaldehyde	C_10_ H_10_ O_3_	7.762	74,281,523.9033	Phenylpropanoid	POS
10	Protocatechuic acid	C_7_ H_6_ O_4_	3.962	72,512,212.0467	Phenolic Acid	POS

## Data Availability

The original contributions presented in this study are included in the article. Further inquiries can be directed to the corresponding author(s).

## Supplementary Materials

The following supporting information can be downloaded at: https://www.mdpi.com/article/10.3390/ph19020194/s1, Figure S1: Cytotoxicity screening of Eucommiae Cortex extract fractions on rat primary tenocytes; Figure S2: Cytotoxicity assessment of aucubin in rat primary tenocytes; Figure S3: Collagenase injection successfully induces a rat tendinopathy model. Table S1: UHPLC gradient elution program; Table S2: Histological grading criteria for tendinopathy (modified Movin score).

## Abbreviations

The following abbreviations are used in this manuscript:

ANOVA	Analysis of Variance
AU	Aucubin
Col I	Collagen Type I
Col III	Collagen Type III
ERβ	Estrogen Receptor β
H&E	Hematoxylin and Eosin
IHC	Immunohistochemistry
IL-1β	Interleukin-1 beta
MDA	Malondialdehyde
MMP-3	Matrix Metalloproteinase-3
MMP-13	Matrix Metalloproteinase-13
ROS	Reactive Oxygen Species
SD	Standard Deviation
SOD	Superoxide Dismutase
TEM	Transmission Electron Microscopy
TNF-α	Tumor Necrosis Factor-alpha
UHPLC-HRMS/MS	Ultra-High Performance Liquid Chromatography coupled with High-Resolution Tandem Mass Spectrometry
