# Supplementary material for: Aucubin from Eucommiae Cortex Alleviates Tendinopathy via an Estrogen Receptor β-Mediated Mechanism
Source: Pharmaceuticals (Basel). 2026 Jan 23;19(2):194. doi: 10.3390/ph19020194 (PMC12943066; doi:10.3390/ph19020194)
Supplement: Supplementary file 1 [file pharmaceuticals-19-00194-s001.zip › pharmaceuticals-4099288-supplementary.pdf]

Supplementary data for

Aucubin from Eucommiae Cortex Alleviates Tendinopathy via an Estrogen Receptor β- Mediated Mechanism

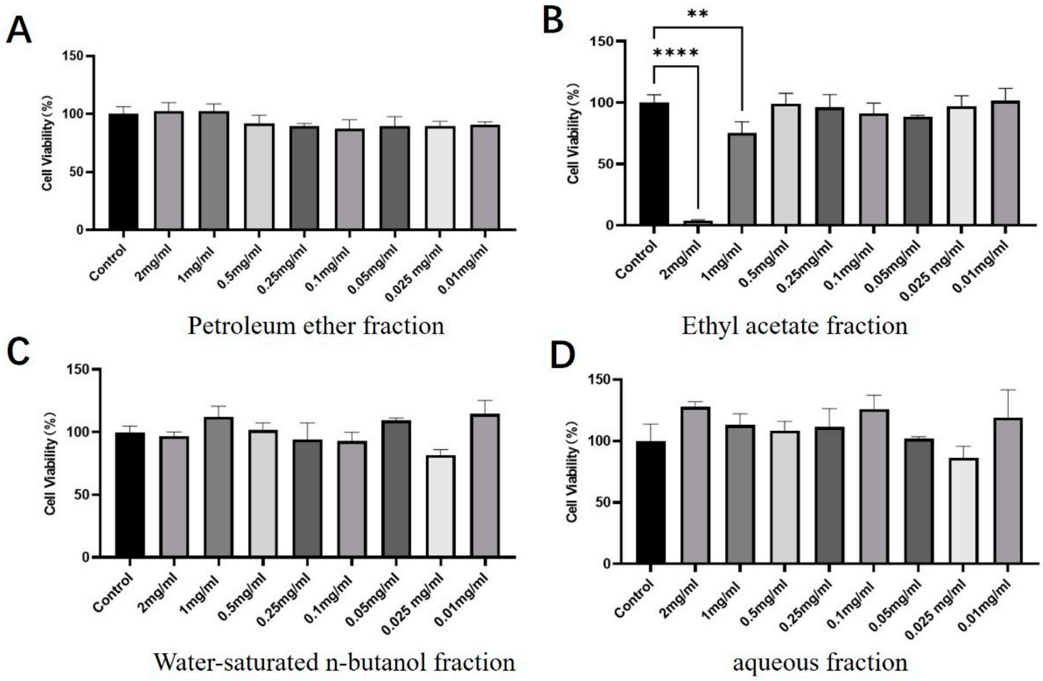

Fig. S1. Cytotoxicity screening of Eucommiae Cortex extract fractions on rat primary tenocytes. Cell viability was assessed after treatment with a range of concentrations of the (A) petroleum ether, (B) ethyl acetate, (C) n-butanol, and (D) aqueous fractions. Data are presented as mean ± SD (n=6). *P < 0.05, **P < 0.01, ***P < 0.001, ****P < 0.0001 vs. Control group.

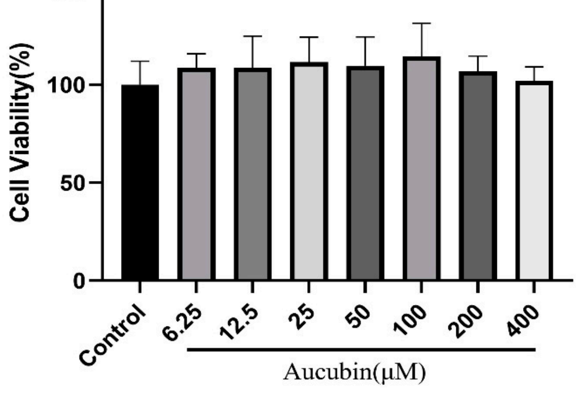

Fig. S2. Cytotoxicity assessment of aucubin in rat primary tenocytes. Cell viability remained unaffected after treatment with a range of concentrations of aucubin for 24 hours. Data are presented as mean ± SD (n=6). *P < 0.05, **P < 0.01, ***P < 0.001, ****P < 0.0001 vs. Control group.

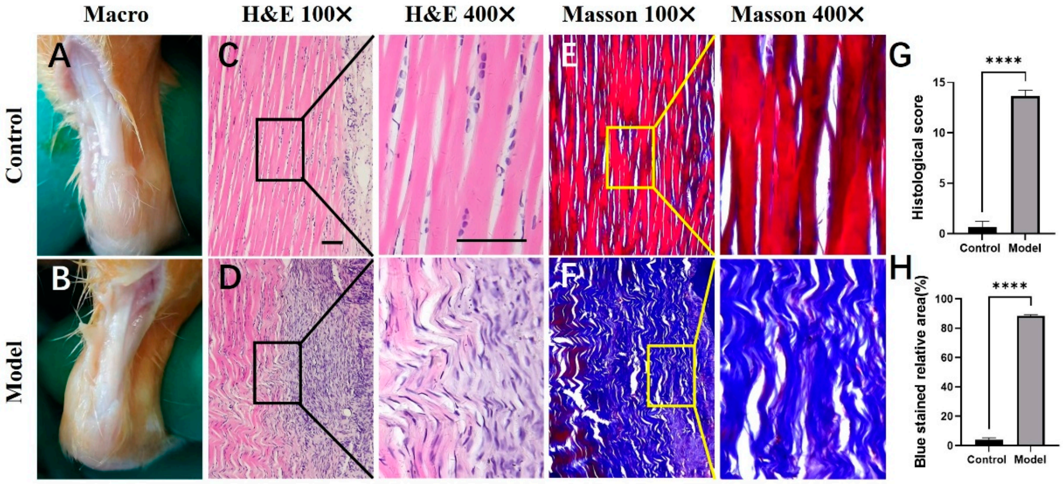

Fig. S3. Collagenase injection successfully induces a rat tendinopathy model. (A, B) Representative macroscopic images of tendons from the (A) Control and (B) Model groups. (C, D) H&E-stained sections show tissue disruption and hypercellularity in the Model group (D) versus Control (C). (E, F) Masson's Trichrome-stained sections reveal disorganized collagen fibers in the Model group (F) compared to the ordered structure in Control (E). (G) Significantly elevated histopathological score based on H&E-stained sections confirms the severity of tendinopathy in the Model group (n=3). (H) Quantification of the blue area in Masson's Trichrome staining indicates a significant increase in immature collagen content in the Model group (n=3). Data are presented as mean ± SD. **P < 0.01, ***P < 0.001, ****P < 0.0001 vs. Control group. Scale bars: 100 μm (C-F).

Table S1. UHPLC gradient elution program.

**Time (min)**	**Flow Rate μL/min)**	**%A (0.1% Formic Acid)**	**%B (Acetonitrile)**
0-2	300	95	5
2-6	300	70	30
6-7	300	70	30
7-12	300	22	78
12-14	300	22	78
14-17	300	5	95
17-20	300	5	95
20-21	300	95	5
21-25	300	95	5

Table S2. Histological grading criteria for tendinopathy (modified Movin score).

	Tendon repair assessment score			
	0	1	2	3
Fiber structure	Continue, long fiber	Slightly fragmented	Moderately fragmented	Severely fragmented
Fiber arrangement	Compacted and parallel	Slightly loose and wavy	Moderately loose, wavy and cross to each other	No identifiable pattern
Rounding of the nuclei	Long spindle shape cells	Slightly rounding	Moderately rounding	Severely rounding
Inflammation (area infiltrated by Inflammation cells)	<10%	10%–20%	20%–30%	>30%
Increased vascularity (area infiltrated by neo-vascular)	<10%	10%–20%	20%–30%	>30%
Cell density	Normal pattern	Slightly increase	Moderately increase	Severely increase
